# Polymorphic Repeat Length in the AIB1 Gene and Breast Cancer Risk in BRCA1 and BRCA2 Mutation Carriers: A Meta-Analysis of Observational Studies

**DOI:** 10.1371/journal.pone.0057781

**Published:** 2013-03-06

**Authors:** Aida Bianco, Barbara Quaresima, Claudia Pileggi, Maria Concetta Faniello, Carlo De Lorenzo, Francesco Costanzo, Maria Pavia

**Affiliations:** 1 Department of Health Sciences, University of Catanzaro Magna Græcia, Catanzaro, Italy; 2 Department of Experimental and Clinical Medicine, University of Catanzaro Magna Græcia, Catanzaro, Italy; University of North Carolina School of Medicine, United States of America

## Abstract

**Objectives:**

We carried out a meta-analysis focusing on the relationship between length of AIB1 gene poly-Q repeat domain as a modifier of breast cancer (BC) susceptibility in patients with BRCA1 and BRCA2 mutation carriers.

**Data sources:**

We searched MEDLINE and EMBASE for all medical literature published until February, 2012.

**Study Eligibility criteria:**

Studies were included in the meta-analysis if they met all the predetermined criteria, such as: (a) case-control or cohort studies; (b) the primary outcome was clearly defined as BC; (c) the exposure of interest measured was AIB1 polyglutamine repeat length genotype; (d) provided relative risk (RR) or odds ratio (OR) estimates and their 95% confidence intervals (CIs).

**Synthesis methods:**

Two of the authors independently evaluated the quality of the studies included and extracted the data. Meta-analyses were performed for case-control and cohort studies separately. Heterogeneity was examined and the publication bias was assessed with a funnel plot for asymmetry.

**Result:**

7 studies met our predetermined inclusion criteria and were included in the meta-analysis. Overall quality ratings of the studies varied from 0.36 to 0.77, with a median of 0.5. The overall RR estimates of 29/29 poly-Q repeats on risk of BC in BRCA1/2, BRCA1, and BRCA2, were always greater than 1.00; however, this effect was not statistically significant. In the meta-analysis of studies reporting the effect of 28/28 poly-Q repeats on risk of BC in BRCA1/2, BRCA1, and BRCA2, the overall RR decreased below 1.00; however, this effect was not statistically significant. Similar estimates were shown for at least 1 allele of ≤26 repeats.

**Conclusions:**

Genotypes of AIB1 polyglutamine polymorphism analyzed do not appear to be associated to a modified risk of BC development in BRCA1 and BRCA2 mutation carriers. Future research on length of poly-Q repeat domain and BC susceptibility should be discouraged and more promising potential sources of penetrance variation among BRCA1 and BRCA2 mutation carriers should be investigated.

## Introduction

Hereditary breast cancer (BC) is characterized by a high degree of clinical and genetic heterogeneity. The inheritance of BC susceptibility in families has led to the identification of two major BC susceptibility genes, BRCA1 and BRCA2 [1]–[3]. Inherited mutations in BRCA1 confer lifetime risks of breast cancer of 70% to 80% [4], [5]. The corresponding risk for BRCA2 mutation carriers is estimated to be 50–60% [6]–[8]. The pathogenic role of nonsense and frame shift mutations is well recognized in breast carcinogenesis, while the impact of missense mutations is still to be defined [9]–[14].

Several studies have reported associations between common polymorphisms in candidate gene studies and the risk of breast or ovarian cancer for mutation carriers, but normally these associations have not been replicated in subsequent studies [15]–[17]. Genes coding for proteins involved in steroid hormone signaling have been examined as risk-modifier candidates. Steroid hormone receptors and their co-activators, such as AIB1, are prime candidate modifiers.

The AIB1 gene coding for a member of steroid hormone receptor coactivator from the SRC1 family of transcriptional co-activators is involved in the control of estrogen-dependent transcription [18], [19]. AIB1, also called SRC3, together with other co-activators and co-repressor proteins is implicated in estrogen signaling pathway and estrogen regulated tumor progression. Within the carboxyl-terminal region of AIB1 lays a polymorphic stretch of glutamine residues (poly-Q) [20] and it has been proposed that AIB1 poly Q domain may directly influence transactivation of estrogen receptor (ER) and thus susceptibility to BC.

Several studies have been conducted in order to analyze length of poly-Q repeat domain as a modifier of BC susceptibility both in patients with sporadic BC [21], [22], and BRCA 1/2 mutation carriers [23]–[29], and the results are yet controversial and inconclusive. Since most studies had relatively small sample sizes and single studies with enough subjects were currently lacking, we may join pieces of evidence from published literature for a meta-analysis. Moreover, this topic has been analyzed in a previous meta-analysis, that demonstrated an increased breast cancer risk in BRCA1/2 mutation carriers for individuals with both alleles C29 polyglutamine repeat [30].

The overarching goal of cancer risk assessment is to indicate cancer risk management recommendations taking into account potential factors that may influence penetrance. Methods to calculate risk make use of health history information, risk factor and family history data in combination with emerging biologic and genetic/genomic evidence to establish predictions. In this study, we carried out a meta-analysis focusing on the relationship between length of poly-Q repeat domain as a modifier of BC susceptibility in patients with BRCA1/2 mutation carriers to provide a basis for more evidence-based counselling and decision making.

## Materials and Methods

### Search Strategy for Identification of Studies

We sought to identify all epidemiological studies that investigated the association between certain polymorphic repeat length in the AIB1 gene and BC risk in BRCA1 and BRCA2 mutation carriers. Studies were identified through electronic databases search and scanning the reference lists of the eligible articles. No restrictions on language were imposed. We searched MEDLINE, and EMBASE for all medical literature published until February, 2012. The search was performed by consecutively entering “BRCA1”, “BRCA2” “mutation”, “mutations”, “carrier”, “carriers”, “breast” “cancer”, and “AIB1”, “NCOA3”, “SRC3” as both medical subject heading terms and text words.

### Inclusion Criteria

Two of the authors were involved in the selection of studies for the review and discrepancies were resolved by discussion after retrieving the full text of articles in question. Studies were included in the meta-analysis if they met all of the following criteria: (a) report original data from case-control or cohort studies; (b) the primary outcome was clearly defined as BC; (c) the exposure of interest measured was AIB1 polyglutamine repeat length genotype (1 allele ≤26, both alleles ≥28, both alleles ≥29); (d) provided relative risk (RR) or odds ratio (OR) estimates and their 95% confidence intervals (CIs) or sufficient data to calculate these estimates; (e) published through February, 2012. If a study appeared in more than one article, data from the most recent publication were used for statistical analysis. We excluded studies including also BRCA1/2 non carriers; letters, abstracts, theses, ecological studies, and conference proceedings; and studies carried out in non-humans.

### Assessment of Study Quality

Two of the authors independently evaluated the quality of the studies included using a modified scoring system that was created on the basis of a systematic review [31] and with reference to QUATSO [32] and STROBE statement for observational studies [33]. Each article was read and scored for quality, and all studies had blinded investigators, institutions, country, and journal. The readers discussed their evaluation, and when discrepancies occurred they were resolved by consensus or, finally, by a third author. The list comprised items felt to be important for the quality of each observational study, including the method for selecting study participants, the adjustment for confounding variables, the method for measuring study variable and design-specific sources of bias, the data analysis, and conflict of interest. The score was calculated as the percentage of applicable quality criteria that were met in each study and a study could range from 0% (none of the quality criterion was met) to 100% (all the quality criteria were met). Studies achieving 67% or more in the score will be regarded as “good” quality; 34–66% “fair”; and, below 33% “poor”. To avoid selection bias, no study was rejected because of these quality criteria.

### Data Extraction

All data from the studies were independently extracted by two of the authors using a designed form. The accuracy of the extracted data was checked by a third reviewer. The following characteristics were recorded from each study: (a) first author’s name, year of publication, and country of the population; (b) study design; (c) number of subjects; (d) confounding factors for matching or adjustment; (e) methods used for collection of data on exposure; (g) RR or OR of BC associated with AIB1 polyglutamine repeat length genotype and the corresponding 95% CI in each subgroup. For the published results of each of the selected studies, data were extracted to allow the calculation of both unadjusted and adjusted ORs with 95% CIs to estimate the association between AIB1 polyglutamine repeat length genotype and the risk of BC.

### Meta-analyses

We planned to analyse case-control and cohort studies separately. The effect on risk for BC was calculated for certain AIB1 polyglutamine repeat length genotype in BRCA1/2 carriers. The analysis was repeated using results for BRCA1 and BRCA2 carriers for those studies where this result was available. Whenever possible, combined risk estimates were calculated by using the risk estimates that reflected the greatest degree of control for other reproductive risk factors (RR or OR adjusted for confounding factors). Heterogeneity among the studies was examined using the method developed by DerSimonian & Laird by calculating the between-study variation based on the *Q* statistic [34]. We considered that there was statistically significant heterogeneity when *p* value was below 0.1 among the results of the included studies. In cases with heterogeneity, we applied random-effects models as opposed to fixed-effect models because they include both within-study sampling error (variance) and between-study variation in the assessment of the uncertainty (CI) of the results of a meta-analysis.

Finally, the presence of publication bias was assessed with a funnel plot for asymmetry, a scatter plot of individual studies that relates the magnitude of the treatment effect against a measure of its precision [35], using for formal statistical testing an adjusted rank correlation test and a regression asymmetry test [36], [37].

All analyses were performed using Stata statistical software (version 11) [38].

## Results

### Study Characteristics

**Figure 1:**
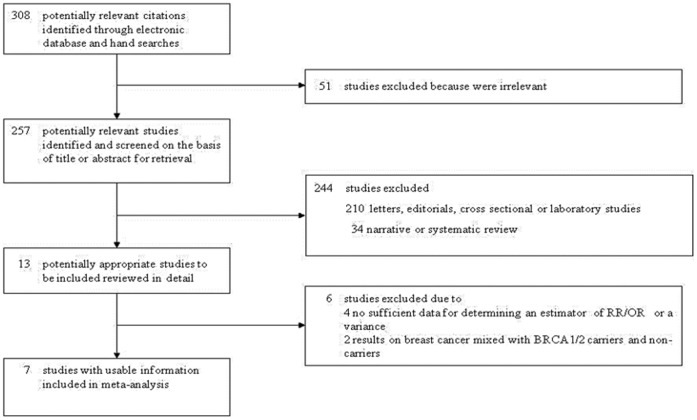
Study selection process and reasons for exclusion of studies.

We identified a total of 308 potentially relevant studies that described the association between certain polymorphic repeat length in the AIB1 gene and BC risk in BRCA1 and BRCA2 mutation carriers, but on obtaining and reading the articles, our predetermined inclusion criteria were met only by 7 studies that were included in the meta-analysis [23]–[29]. A list of the excluded papers is available from the authors. Articles were excluded from the analyses for any one of the following reasons: (1) review paper; (2) letters or editorial; (3) laboratory study; (4) survey study; (5) no sufficient published data for determining an estimator of RR (OR) or a variance; (6) results on BC were mixed with BRCA1/2 mutation carriers and non-carriers (Figure 1). The overall agreement among reviewers on selection of the studies was excellent, since it was higher than 99%.

The summary characteristics of all studies included in the meta-analysis are described in **Table 1**. The sample size of the 7 included studies (5 cohort and 2 case-controls) varied between 57 and 642 for exposed and between 2 and 1,407 for non-exposed in cohort studies, respectively, and between 278 and 319 for cases and between 170 and 290 for controls in case-control studies, respectively. The studies were geographically heterogeneous: 5 studies involved European samples, 4 studies were conducted in North America, 1 in Israel and 1 in Australia. To give an indication of the actual RR/OR found in the studies, we also show for each study the relative risks for the group with the different exposure in that study. Four studies for AIB1 polyglutamine repeat length genotype (1 allele ≤26, both alleles ≥28, both alleles ≥29) reported adjusted RR/ORs [25], [26], [28], [29]; two studies provided sufficient data to calculate a crude RR [24], [27], and the paper by Rebbeck and colleagues [23] reported adjusted OR for both alleles ≥28 and ≥29 length genotype groups, and sufficient data to calculate a crude OR for 1 allele ≤26 length genotype group. Three papers provided information on BRCA1 and BRCA2 carriers separately and combined, two papers only on BRCA1 carriers, one paper on BRCA1 and BRCA2 carriers separately, and another paper only the combined data. Two studies did not use statistical adjustment for any potential confounding factors. Five papers made some adjustments for potential confounding factors; only two, however, adjusted for year of birth, age at menarche, parity, age at first live birth and oral contraceptive use. Studies using both polymerase chain reaction (PCR)-amplified and non-amplified genotyping methods were included. There were no restrictions on PCR primers’ utilization.

**Table 1:** Characteristics of observational studies of the relation between polymorphic repeat length in the AIB1 gene and breast cancer risk in BRCA1 and BRCA2 mutation carriers included in the meta-analysis.

Authors	Country	Studytype	No. of cases/exposed	No. of control/not exposed	Variables of adjustment	Mutationcarriers	AIB1 polyglutamine repeat length genotype groups	Crude (c) or adjusted (a)RR/OR estimate(95% CI)	Quality score
Rebbeck et al.,2001 [23]	USA, Canada	Case-control	278	170	Age, year of birth, age at menarche, parity, age at first live birth, smoking status	BRCA1/2	1 allele ≤26	0.64 (0.41–1)^c^	0.64
							both alleles ≥28	1.59 (1.03–2.47)^a^	
							both alleles ≥29	2.85 (1.64–4.96)^a^	
Kadouri et al.,2004 [24]	Israel, UK	Cohort	195	116	None	BRCA1/2	1 allele ≤26	0.87 (0.7–1.09)^c^	0.58
							both alleles ≥28	1.15 (0.92–1.44)^c^	
							both alleles ≥29	1.21 (1.01–1.46)^c^	
			138	84		BRCA1	1 allele ≤26	0.77 (0.56–1.04)^c^	
							both alleles ≥28	1.31 (0.96–1.78)^c^	
							both alleles ≥29	1.21 (0.98–1.51)^c^	
			57	31		BRCA2	1 allele ≤26	1.07 (0.77–1.49)^c^	
							both alleles ≥28	0.93 (0.67–1.3)^c^	
							both alleles ≥29	1.23 (0.83–1.81)^c^	
Hughes et al.,2005 [25]	France, Greece, USA	Cohort	642	449	Year of birth, parity	BRCA1/2	both alleles ≥28	0.88 (0.75–1.04)^a^	0.5
							both alleles ≥29	1.06 (0.88–1.27)^a^	
						BRCA1	both alleles ≥28	1.02 (0.83–1.26)^a^	
							both alleles ≥29	1.02 (0.81–1.28)^a^	
						BRCA	both alleles ≥28	0.67 (0.51–0.88)^a^	
							both alleles ≥29	1.16 (0.86–1.57)^a^	
Spurdle et al.,2006 [26]	UK, Australia, North America, Quebec	Cohort	598	492	Year of birth, age at menarche, parity, oral contraceptive use, ethnicity	BRCA1	both alleles ≥28	0.76 (0.59–0.97)^a^	0.5
							both alleles ≥29	0.95 (0.73–1.24)^a^	
			392	269		BRCA2	both alleles ≥28	1.34 (0.92–1.93)^a^	
							both alleles ≥29	1.18 (0.82–1.7)^a^	
Colilla et al.,2006 [27]	USA, Canada	Cohort	176	1407	Year of birth, age at menarche, parity, age at first live birth, menopausal status, height, smoking status, oral contraceptive use, ethnicity	BRCA1	1 allele ≥26	1 (0.8–1.26)^c^	0.42
							both alleles ≥28	1 (0.88–1.13)^c^	
							both alleles ≥29	1.05 (0.84–1.33)^c^	
Jakubowska et al.,2010 [28]	Poland	Case-control	319	290	Year of birth, age at menarche, parity, age at first live birth, breastfeeding, BMI*, smoking status, oral contraceptive use	BRCA1	both alleles ≥28	0.87 (0.54–1.41)^a^	0.77
Kleibl et al.,2011 [29]	Czech Republic	Cohort	211	32	None	BRCA1/2	1 allele ≤26	0.96 (0.85–1.09)^a^	0.36
							both alleles ≥28	0.92 (0.66–1.28)^a^	
							both alleles ≥29	0.93 (0.69–1.26)^a^	
			148	30		BRCA1	1 allele ≤26	0.98 (0.84–1.14)^a^	
							both alleles ≥28	0.9 (0.61–1.32)^a^	
							both alleles ≥29	1.08 (0.76–1.53)^a^	
			63	2		BRCA2	1 allele ≤26	0.95 (0.75–1.2)^a^	
							both alleles ≥28	1.09 (0.57–2.11)^a^	
							both alleles ≥29	0.66 (0.37–1.18)^a^	

* BMI: body mass index.

### Data Quality

The overall agreement among reviewers on evaluation of the quality of the studies was higher than 85% and differences were resolved by a third reviewer.

The potential risk of bias associated with various aspects of study design is described in **Table 2** that summarizes the quality of the epidemiological studies included in the meta-analysis. Overall quality ratings of the studies varied from 0.36 to 0.77, with a median of 0.5. In all studies cases and controls were identified without knowledge of exposure status, controls were drawn from the same population of cases and basic sample characteristics were presented. All cohort studies retrospectively ascertained cohort and in 60% of them it was not specifically indicated in the text that exposed/non-exposed subjects were identified without knowledge of disease status. The study of Rebbeck and colleagues [23] generated a nested case-control sample using an incidence density sampling design. None of the studies specified cancer diagnosis criteria although diseases like BC are subject to relatively little misclassification (false negatives are unlikely given the severity of the disease, and false positives are unlikely given the medical scrutiny of suspected cases).

**Table 2:** Items used in quality scoring for studies of the association between polymorphic repeat length in the AIB1 gene and breast cancer risk in BRCA1 and BRCA2 mutation carriers.

Quality scoring item	% of studies complying^a^
**Case-control studies**	
Cases either randomly selected or selected to include all cases in a specific population	100
Cases identified without knowledge of exposure status	100
Controls drawn from the same population of cases	100
No known association between control status and exposure	100
**Cohort studies**	
Comparison/Description of persons who did and did not participate	20
Comparison of who were and were not lost to follow-up	0
Exposed/non-exposed subjects identified without knowledge of disease status	40
**All studies**	
Any response rate was reported	0
An estimation of the sample size was made	14.3
*Adjustment or matching for confounders*	
Year of birth	71.4
Age at menarche	57.1
Parity	71.4
Age at first live birth	42.9
Smoking status	42.9
Oral contraceptive use	42.9
Ethnicity	28.6
Menopausal status	14.3
Height	14.3
Breastfeeding	14.3
Body mass index (BMI)	14.3
*Statistical methods*	
Basic characteristics listed	100
Losses of participants, missing data or other design defects were adequately treated	0
Precise p values and/or confidence interval and/or power given	100
*Conflict of interest declared*	42.9

a If compliance is not specifically indicated in the text, non compliance is assumed.

The extent of adjustment for potential confounding factors in the relationship between certain polymorphic repeat length in the AIB1 gene and BC risk in BRCA1 and BRCA2 mutation carriers varied across studies. 71.4% of studies adjusted for year of birth and parity, 57.1% for age at menarche, but 14.3% for the menopausal status, height, breastfeeding and body mass index. Only 2 studies presented adjusted OR for ethnicity. Information bias associated with failure to consider ethnicity as a confounding variable may have been a problem in the study of Kadouri and colleagues [24] that assessed the effect of the polyglutamine repeat polymorphism in the AIB1 gene on BC risk in BRCA1 and BRCA2 mutation carriers, mainly of Ashkenazi origin. No studies reported any response rate and adequately treated losses of participants, missing data or other design defects. Three studies declared conflict of interest.

### Meta-analysis

**Figure 2:**
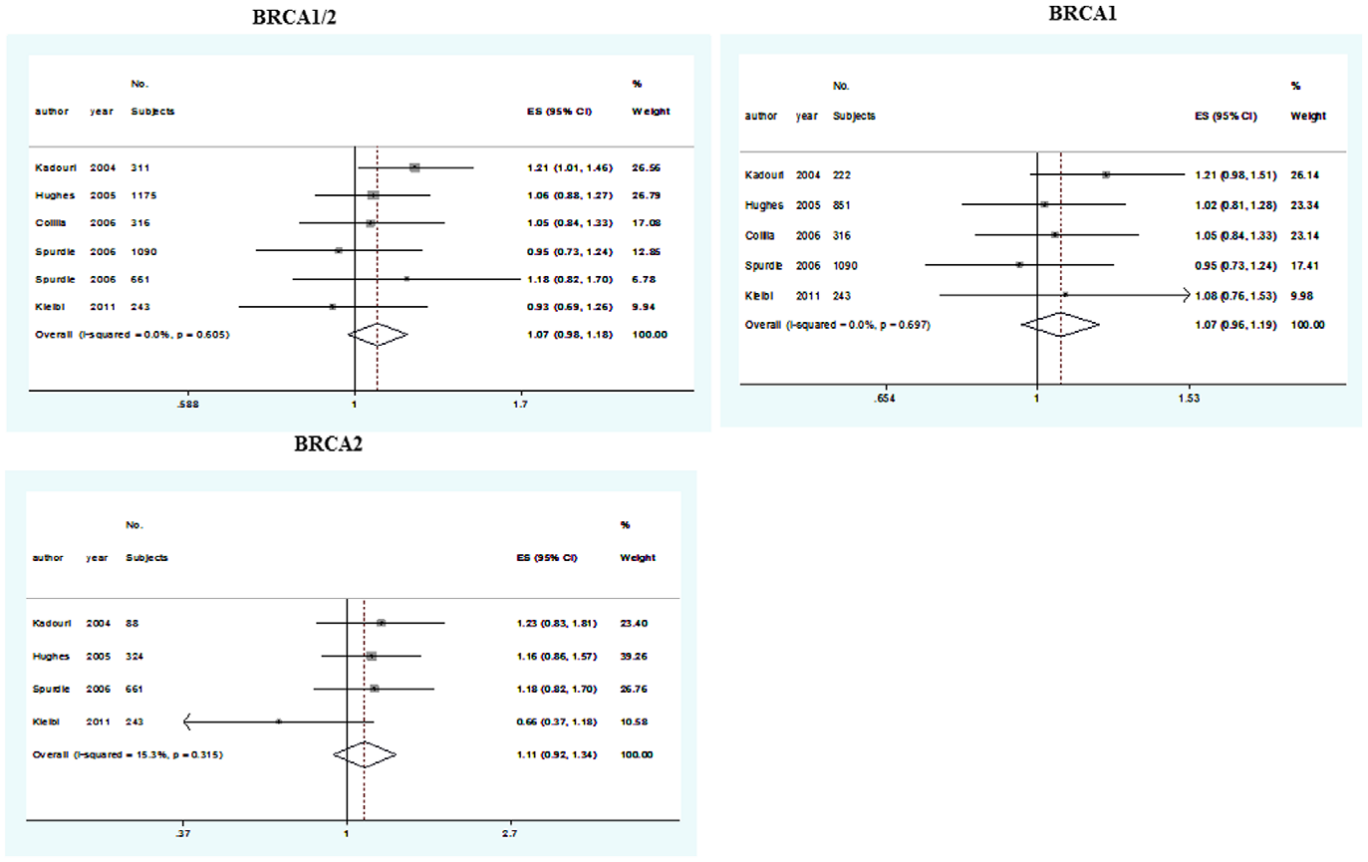
Meta-analysis exploring the effect of 29/29 poly-Q repeats on risk of BC in BRCA1/2, BRCA1, and BRCA2 mutation carriers.

When all extracted data were pooled, 5,980, 6,589 and 3,138 patients were eligible for analysis on 29/29 poly-Q repeats, 28/28 poly-Q repeats and at least 1 allele of ≤26 repeats, respectively. The objectives of all available studies were to analyze the effect of AIB1 poly-Q domain polymorphism genotypes and BC onset among BRCA1/2 mutation carriers. **Figure 2** shows data of meta-analysis exploring the effect of 29/29 poly-Q repeats on risk of BC in BRCA1/2, BRCA1, and BRCA2, respectively. The overall RR estimates of these meta-analyses were always greater than 1.00, indicating a potential effect of 29/29 poly-Q repeats on BC onset, however, this effect was not statistically significant.

**Figure 3:**
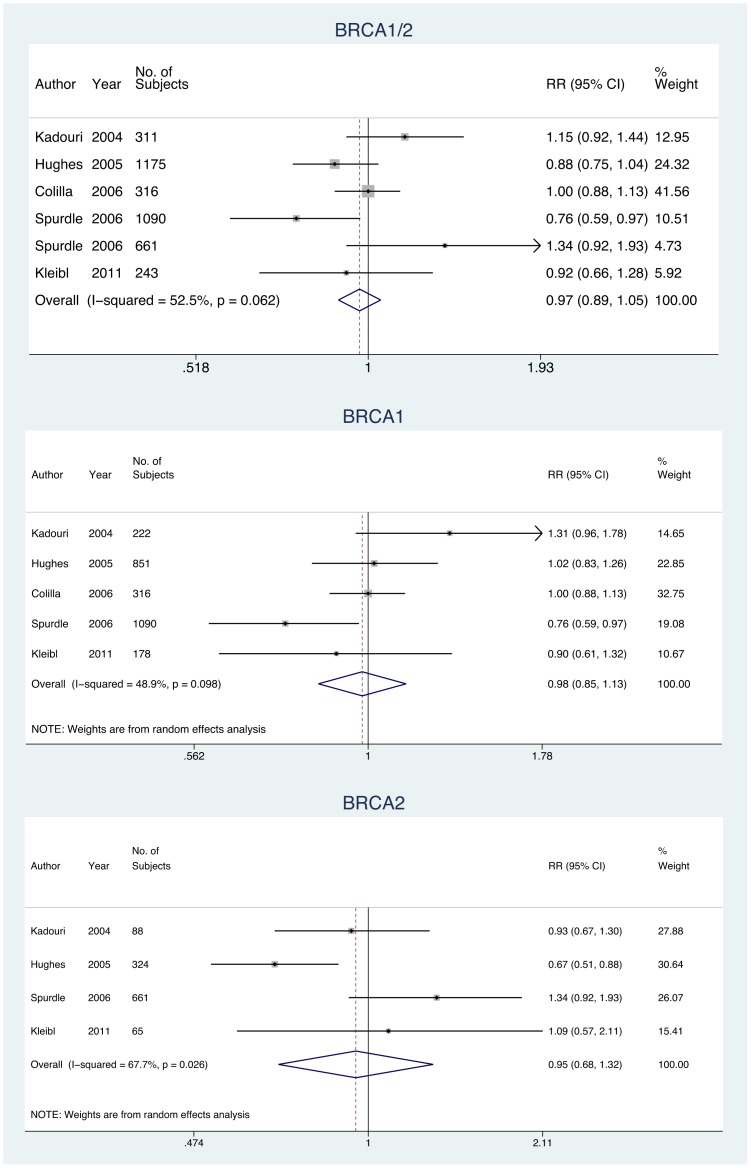
Meta-analysis exploring the effect of 28/28 poly-Q repeats on risk of BC in BRCA1/2, BRCA1, and BRCA2 mutation carriers.

**Figure 4:**
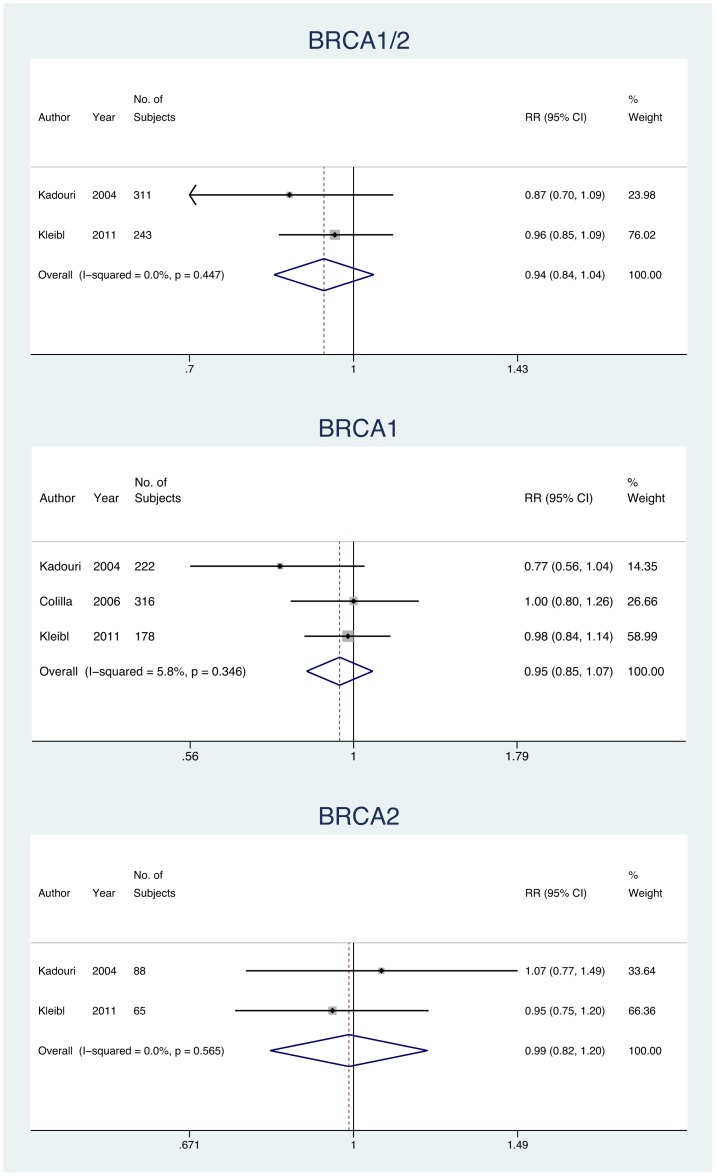
Meta-analysis exploring the effect of 26/26 poly-Q repeats on risk of BC in BRCA1/2, BRCA1, and BRCA2 mutation carriers.

In the meta-analysis of studies reporting the effect of 28/28 poly-Q repeats on risk of BC in BRCA1/2, BRCA1, and BRCA2, the overall RR decreased below 1.00, however, this effect was not statistically significant **(** **Figure 3** **)**. The pooled RR estimate for at least 1 allele of ≤26 repeats performed in BRCA1/2, BRCA1, and BRCA2 mutation carriers were similar to the 28/28 poly-Q repeats **(** **Figure 4** **)**. The *Q* statistic test of homogeneity found a statistically significant heterogeneity across the studies on 28/28 poly-Q repeats; indeed, the results for BRCA1 (*Q*  = 7.83, df  = 4, *p = *0.098) and for BRCA2 carriers (*Q*  = 9.29, df  = 3, *p = *0.026) were heterogeneous.

A separate meta-analysis was performed for the two case-control studies reporting data on 28/28 AIB1 poly-Q domain in BRCA1/2 and BRCA1 mutation carriers, respectively. The overall OR resulting from those studies was greater than 1.00, however, this effect was not statistically significant (data not shown).

Funnel plots displaying RRs of the individual study versus the reciprocal of their standard errors did not show any substantial asymmetry in studies exploring the effect of 29/29 and 28/28 AIB1 poly-Q domain on risk of BC in BRCA1/2 (*p* = 0.117, Begg & Mazumdar adjusted rank correlation test, *p* = 0.405, Egger et al. regression asymmetry test; *p* = 0.766, Begg & Mazumdar adjusted rank correlation test, *p* = 0.602, Egger et al. regression asymmetry test, respectively). Analogously, no significant funnel plot asymmetry was observed for studies exploring the effect of at least 1 allele of ≤26 repeats on risk of BC in BRCA1/2, using the test of Egger et al. (*p* = 0.372), as well as the test of Begg & Mazumdar (*p* = 0.317).

## Discussion

Several studies have reported associations between common polymorphisms in candidate genes studies and the risk of BC for mutation carriers. Steroid hormone receptors and their co-activators, such as AIB1, have been prime examined as risk-modifier candidates. A few studies have so investigated the genetic contribution of the AIB1 gene polyglutamine repeat length as a risk factor influencing BC onset in BRCA1/2 carriers with contradictory results. Hence, we conducted a meta-analysis of observational studies examining the association between certain polymorphic repeat length in the AIB1 gene and BC risk in BRCA1 and BRCA2 mutation carriers. Despite AIB1 is considered to be only a low penetrant BC modifier, its clinical role could be potentially considerable because the population frequency of AIB1 genotype coding for 28/28 poly-Q repeats is substantially higher (10%) compared to the majority of other low penetrant BC alleles.

Meta-analysis performed did not reveal any association between certain polymorphic repeat length in the AIB1 gene and BC risk in BRCA1 and BRCA2 mutation carriers. The number of analyzed BRCA2 mutation carriers in the included studies is limited, but the overall RR estimates of the meta-analyses of all AIB1 poly-Q domain polymorphism genotypes were always not statistically significant. Moreover, we also performed separate meta-analyses for BRCA1 and BRCA2 mutation carriers since we believe that pooling of BRCA1 and BRCA2 mutation carriers for analyses of AIB1 poly-Q repeat polymorphism could be disputable considering the substantial differences in effects of AIB1 polymorphism in these groups and also assuming the diverse histopathological and molecular characteristic of breast tumors in BRCA1 and BRCA2 carriers.

The initial studies of Rebbeck et al. [23] and Kadouri et al. [24] reported the positive correlation between BC risk and increased lengths in AIB1 poly-Q repeat notably in BRCA1 mutation carriers. Later studies failed to confirm the association of AIB1 poly-Q repeat length polymorphism with BC risk in BRCA1/2 mutation carriers. A more recent study [29] indicated that carriers of the AIB1 genotype coding for 28/28 poly-Q repeats with a mutation in the BRCA1 gene had reduced BC risk compared to BRCA1 mutation carriers with other AIB1 genotypes, confirming findings from a previous study [27]. Moreover, the authors observed that AIB1 28/28 genotype strongly increased the BC risk only in carriers of BRCA2 mutation localized in exon 11. Studies that performed additional analysis evaluating the BC risk in women carrying at least 1 allele of ≤26 repeats in the polymorphic region of AIB1 found no significant differences on risk of BC in BRCA1/2 mutation carriers. Only the study by Kadouri et al. indicated a significant reduction of BC risk in carriers of BRCA1 mutations with the shorter polyglutamine chain of AIB1 gene [24].

A recent meta-analysis presented subgroup analyses considering BRCA1 and BRCA2 mutation carriers and found increased breast cancer risk for women with both alleles ≥29 repeat [30]. The comparison with our results is difficult, regardless of the differences in the inclusion criteria used. In particular, the meta-analysis by Zhang et al. involved 2 of the cohort [24], [29] and 2 of the case-control [23], [28] studies included in ours and 1 case-control study [22] that we excluded because reported the estimates of BC risk and allele frequency only among cases carrying a germline mutation in BRCA1 and BRCA2 and not for controls. The objective of the present meta-analysis was to combine all available information, which yields an increase in power, to generate an integrated result used to provide evidence for genetic counselling strategy in specific subgroups of population with an increased risk of BC and to design future research.

Moreover, we performed separate meta-analyses for cohort and case-control studies to analyze the association between AIB1 polymorphisms and BC susceptibility in BRCA1 and BRCA2 mutation carriers.

On the other hand, differences in the associations of the modifying polymorphisms with breast cancer risk for BRCA1 and BRCA2 mutation carriers are likely to reflect differences in the biology of tumor development in these two groups of women at high risk of BC.

In this meta-analysis the quality of the observational studies was fair. The evaluation of the quality of the studies in a meta-analysis may contribute to point out limitations in published studies and to suggest ways to improve the methodology of studies in further research. For example, only three studies declared conflict of interest and, since funding source has been shown to be an important source of heterogeneity, the sponsoring organization should be disclosed, so any effect on analysis could be examined. Indeed, it is reported that meta-analyses without (or undeclared) financial support are of inferior quality than meta-analyses with profit and non-profit support [39].

The value of the current meta-analysis compensates for the individual lack of precision of most studies, a problem alleviated by pooling. As non significant results could lead to incorrectly accept a false null hypothesis, making a type II error of omission, we performed a post-hoc power calculation to examine whether the lack of adequate power accounts for the blurring of associations. The post-hoc power calculation showed that our set of studies had sufficient statistical power (0.85) to detect a significant difference between the effect size in the two groups of d  = 0.14.

### Strengths and Limitations of the Study

The main strengths of our study include the rigorous methods employed to identify studies and assessment of potential risk of bias. The lack of association between certain polymorphic repeat length in the AIB1 gene and BC risk in BRCA1 and BRCA2 mutation carriers was robust to a number of subgroups. We did not observe any evidence of publication bias in the studies included in our review, which decreases the likelihood that our findings were related to our method of selecting articles. Moreover, the amount of heterogeneity among studies seems to be low and the between-studies dispersion seems less than would be expected by chance.

The strengths of the present meta-analysis also include absence of restriction to studies published in English, since language restriction could introduce bias in the results of the meta-analysis [40], [41].

Possible limitations of all meta-analyses of observational studies are related to absence of a “gold standard” instrument for the quality assessment and, although several assessment scales and checklists have been used [42]–[44] none of them has been fully validated. Moreover, one of the most well-known proposed scoring system, the Newcastle-Ottawa-Scale (NOS) by Wells [45] et al. has recently been considered of unknown validity at best, or including quality items that are even invalid [46]. Therefore, we preferred to propose a modified scoring system that, with reference to QUATSO [32] and STROBE statement for observational studies [33], appeared to us more adequate to address the specific methodological issues related to our study.

The present meta-analysis include poor methodological quality of the studies on which the analysis is based. Many of the studies included in our review were at some potential risk of bias from certain aspects of study design and the potential biases inherent in all observational studies may have contributed to the observed findings. It has been argued that since meta-analysis of observational studies may produce very precise, but spurious results, statistical combination of these data should not be the prominent component. Therefore, taking the quality of studies into account in a meta-analysis has the potential to enhance the validity of a meta-analysis because quality is implicitly a measure of validity [47]. Moreover, the evaluation of the quality of the studies in a meta-analysis may contribute to point out limitations in published studies and suggest ways to improve the methodology of studies in further research. In this meta-analysis the quality of the observational studies, in particular cohort studies, was scant with regard to the various methodological aspects of the study design (for example, comparison of who were and were not lost to follow-up, comparison or description of persons who did and did not participate, knowledge of disease status after exposed/non-exposed subjects were identified). Other shortcomings were related to the response rate reporting and adjustment for confounders. Many but not all of the studies adjusted for potential confounding factors, although not all potential confounders were adjusted for in every study, and this might have had an impact on our overall dataset. In our meta-analysis we included the most fully adjusted hazard ratio presented in the articles. Indeed, only 2 of the 7 included studies adjusted for what we considered in our quality assessment tool as essential confounders (year of birth, age at menarche, parity, age at first live birth, smoking status, oral contraceptive use).

In conclusion, on the basis of epidemiological evidence, genotypes of AIB1 polyglutamine polymorphism –analyzed in categories according to cut-points 1 allele ≤26, both alleles ≥28 and ≥29- do not appear to be associated to a modified risk of BC development in BRCA1 and BRCA2 mutation carriers. Future research on length of poly-Q repeat domain and BC susceptibility should be discouraged and more promising potential sources of penetrance variation among BRCA1 and BRCA2 mutation carriers should be investigated.
